# Enhancing Piezoelectricity of Polyacrylonitrile–Cellulose Composite Nanofibers via Zigzag Conformation

**DOI:** 10.3390/polym17040465

**Published:** 2025-02-10

**Authors:** Joong Yeon Lim, Won Suk Kwak, Minwook Park, Young Seong Kim

**Affiliations:** Department of Mechanical, Robotics and Energy Engineering, Dongguk University, Jung-gu, Seoul 04620, Republic of Korea; jylim@dongguk.edu (J.Y.L.); danny113@dgu.ac.kr (W.S.K.); mwp4434@dgu.ac.kr (M.P.)

**Keywords:** polyacrylonitrile, cellulose nanocrystal, composite, piezoelectric, zigzag confirmation

## Abstract

A novel piezoelectric material, polyacrylonitrile (PAN) nanofibers, exhibits significant piezoelectric effects when a high content of planar zigzag structures is present. To enhance the contribution of planar zigzag structures to energy conversion while preserving the structure of PAN nanofibers, a novel approach was developed to increase planar zigzag content by incorporating cellulose nanocrystals (CNCs) rather than modifying conventional synthesis conditions. In this study, CNCs were introduced during the electrospinning process of PAN formation, and the increased planar zigzag content was confirmed through X-ray diffraction (XRD), electrical characterization, and Fourier transform infrared spectroscopy (FTIR) analyses. This study, for the first time, demonstrates that CNC addition to PAN enhances the mechanical properties and piezoelectric performance by promoting the formation of zigzag structures, which play a crucial role in the piezoelectric effect. The PAN–CNC composite holds great potential for applications in new piezoelectric devices. With CNC incorporation, the voltage increased by 68.9%, and the current increased by 80% compared to regular PAN. The generated energy is suitable for human applications and can also power commercial devices, making these findings pivotal for the advancement of piezoelectric materials and devices.

## 1. Introduction

In the fields of IoT and low-energy applications, functional polymers are being extensively researched, and self-powered systems are gaining significant attention. Regarding energy harvesting, studies on piezoelectric and conductive polymers have been conducted, along with comparative research on their mechanoelectrical conversion properties [1,2]. In particular, for polymer fabrication in energy-related applications, 3D printing and electrospinning methods are widely utilized [3,4]. The 3D printing technique allows for the fabrication of free-form polymers and enables the production of polymers in specific phases [5]. Meanwhile, the electrospinning method is a versatile approach for forming ultrathin nanofibers, providing multifunctional properties [6].

Piezoelectric polymers have been widely utilized recently due to their flexibility and sensitivity to pressure variations. These piezoelectric polymers offer significant advantages, particularly when integrated into wearable devices utilizing biocompatible components, making them suitable for sensor module applications [7]. Through the electrospinning process, piezoelectric polymers can be fabricated into nanofibers and subsequently into electronic textiles, which is a widely adopted technique. This approach allows for the uniform synthesis of fiber structures, which has found extensive applications in the field of electronic textiles [8,9]. Additionally, it is evident that these technologies are being employed in the development of smart wearable devices [10,11].

Piezoelectric polymers can be categorized into amorphous polymers and semi-crystalline polymers based on their physical structure. However, these polymers share common characteristics related to the molecular dipole properties of their molecules. One notable feature is that when the polarization in the direction of the dipole becomes dominant, a larger dipole moment is generated, which in turn leads to greater piezoelectricity [12].

Recently, polyacrylonitrile (PAN) has gained attention as a promising piezoelectric polymer. As an amorphous vinyl-type polymer, PAN exhibits excellent piezoelectric performance and mechanical properties due to its high dipole -CN (cyano) groups [13,14,15].

An important characteristic of PAN is that its piezoelectric performance varies depending on the orientation of its -CN groups. In the solid state, PAN exhibits two primary structures: planar zigzag and 3^1^-helical [16,17,18]. While initially known for having lower piezoelectric properties compared to polyvinylidene fluoride (PVDF), recent studies have demonstrated that PAN nanofiber membranes fabricated through electrospinning can exhibit higher piezoelectric performance than PVDF. This improvement is attributed to the increase in planar zigzag structures within PAN’s polymer structure, which varies across forms such as powder, cast film, and nanofibers [12].

Furthermore, the planar zigzag structure of PAN has been shown to increase in composite polymers, particularly through the incorporation of highly aligned cellulose nanocrystals, enabling enhanced piezoelectric performance [19]. Based on its excellent mechanical properties, PAN has been extensively studied as a precursor for carbon fibers [20,21,22]. The synthesis of PAN with the addition of novel human-friendly materials has recently garnered attention for the emergence of new properties.

Cellulose nanocrystals (CNCs) possess advantages such as hydrophilicity and biocompatibility, making them widely used as reinforcing agents in polymer composites to enhance mechanical properties. They are well known for their ability to form networks when incorporated into polymer matrices [23]. A major challenge, however, is achieving a homogeneous distribution of CNCs within the polymer. The electrospinning method effectively addresses this challenge by ensuring well-dispersed CNCs within the polymer matrix [24]. Notably, the PAN–CNC composite has demonstrated its potential for biomedical applications, including its use as a composite material in dental applications [25].

In this study, for the first time, a composite of PAN and CNC was fabricated to enhance planar zigzag structures, and a piezoelectric device was developed using the electrospinning method to analyze the properties of the PAN polymer composite. Specifically, the changes in nanofiber morphology and the increase in planar zigzag formation due to the addition of CNC were examined, along with the resulting improvement in electrical characteristics. The ability to easily control the increase in planar zigzag structures using PAN–CNC composites presents limitless potential for new piezoelectric device applications.

## 2. Materials and Methods

### 2.1. Materials

The PAN and PAN–CNC composites were synthesized using the following reagents and chemicals: N,N-Dimethyl formamide (DMF, 99.8%) from Daejung Chemicals (Siheung, Republic of Korea), polyacrylonitrile (PAN) (average molecular weight M_v_ 150,000) from Sigma-Aldrich (St. Louis, MO, USA), and cellulose nanocrystal (CNC, 10.4%) from The University of Maine (Orono, ME, USA).

### 2.2. Synthesis of Composite Material Solution

PAN powder and CNC powder were prepared, and CNC, at a predetermined weight percent, was added to 60 mL of DMF. Based on 60 mL of DMF, PAN was prepared at a concentration of 10 wt%, while CNC was incorporated at 1, 2, and 3 wt%. The mixture was subjected to ultrasonication until the CNCs were no longer visible, ensuring proper dispersion. Subsequently, the beaker was placed on a heating mantle set at 50 °C, and PAN powder was gradually added while stirring. Stirring was maintained at 500 rpm for 24 h to ensure thorough mixing. Ultrasonication was not performed when CNC was not added. The synthesis temperature was specifically set to facilitate the transformation of PAN’s molecular structure into a zigzag configuration.

### 2.3. Eletrospinning

**Figure 1:**
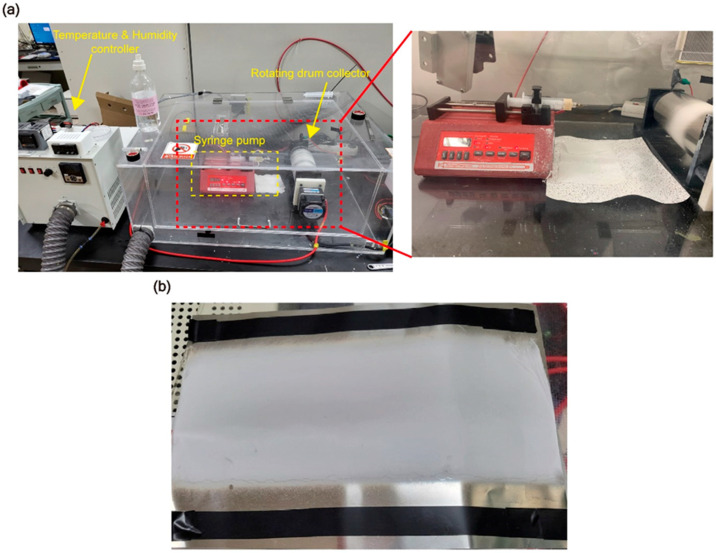
(**a**) Experimental setup. (**b**) Nanofiber membrane of the PAN composite.

Electrospinning was conducted in an acrylic chamber with controlled temperature and humidity set to 27 ± 1 °C and 45 ± 2%, respectively. Using a syringe pump, a flow rate of 1 mL/h was maintained, and 2 mL of solution was electrospun over 2 h. A 22G syringe needle was used, with the distance between the needle and a drum collector (9 mm in diameter and 20 cm in length) set to 15 cm. The drum collector was rotated at 300 RPM. A high-voltage power supply (SJ-5001P, SEJIN ELECTRONICS CO., Seoul, Republic of Korea) applied a voltage of 18.5 kV during the process (Figure 1a).

After electrospinning, the membranes were dried in a vacuum oven at 50 °C for 10 h to completely remove any residual solvent and DMF (Figure 1b).

### 2.4. Fabrication of Piezoelectric Device

The PAN membrane was sampled into 4 cm × 4 cm pieces, and copper electrodes (3 cm × 2 cm) were placed in a sandwich configuration on the PAN membrane. The device was then encapsulated using a 5 cm × 5 cm polyethylene terephthalate (PET) film. Each copper electrode was extended with additional connections, measuring 0.5 cm × 6 cm, for electrical signal measurements.

### 2.5. Characterization

A Mini SmartShaker (K2007E01, The Modal Shop, Inc., Cincinnati, OH, USA) was used with a Function/Arbitrary Waveform Generator (UTG1010A, UNI-T, Dongguan, Guangdong, China) set to a frequency of 10 Hz and an amplitude of 1 Vpp. Voltage and current measurements were performed using a Digital Multimeter (DMM6500, Keithley, Solon, OH, USA). The force was measured using a Digital Force Gauge (AMF-500, ALIYIQI, Yueqing, Zhejiang, China).

XRD analysis was conducted using an X-ray Diffractometer (Ultima IV, Rigaku, Japan), and topology measurements were performed using Scanning Electron Microscopy (SEM) (JSM-7800F Prime, JEOL Ltd., Tokyo, Japan) with a 5 kV accelerating voltage and a lower-electron detector. Fourier Transform Infrared (FTIR) spectrum analysis was carried out using a Functional Anal-ATR-FTIR Spectrometer (IdentifyIR, Smiths Detection, London, UK).

Fiber diameter analysis through SEM was conducted using ImageJ software to measure the diameters of the fibers.

## 3. Results and Discussion

**Figure 2:**
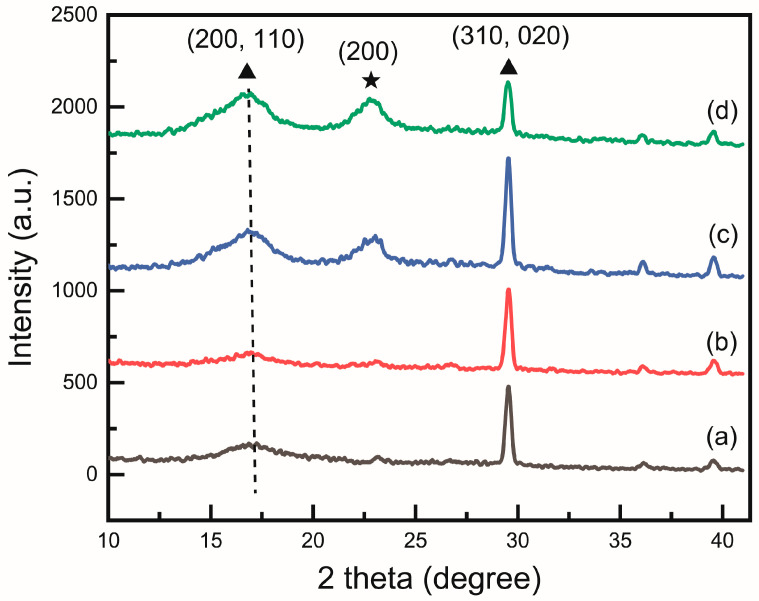
X-ray diffraction (XRD) pattern of (**a**) PAN, (**b**) PAN/CNC1, (**c**) PAN/CNC2, and (**d**) PAN/CNC3. Triangles represent the crystalline planes of PAN, while star symbols indicate the crystalline planes of CNC. The dotted line indicates the position of the shifted peak in (**d**).

The XRD analysis in Figure 2 confirms the successful formation of the PAN and CNC composite. A semicrystalline polymer peak of PAN can be observed at 16.8°, while the peak at 22.8° increased with the addition of CNC. Although the peak was very weak at 1 wt%, it became clearly visible from 2 wt%, indicating that CNCs were forming along the (200) plane. The leftward shift of the 16.8° peak due to CNC addition indicates an increased incorporation of the zigzag conformation. The leftward shift of the 16.8° peak due to CNC addition indicates an increased incorporation of the zigzag conformation. Previous studies have compared casting films, powders, and nanofibers, confirming that nanofibers exhibit the lowest zigzag content [12].

The peaks near 36° and 40° are typical meridional peaks observed in PAN, corresponding to the planar zigzag and helical sequences, respectively. These peaks are observed due to the repetitive axial structure of the PAN polymer chains [26,27]. This indicates that the C-C bond distance in the main chain of PAN becomes shorter, which is slightly influenced by the molecular conformation of the polymer [28]. In the planar zigzag structure, the C-C bond distance in PAN is shorter compared to the helical structure, causing the electron cloud to shift toward the carbon atoms. This results in a high-field chemical shift of the carbon atoms due to the squeeze effect of the -CN nitrogen atoms [12].

Our data show that as the CNC wt% increased, a shift toward lower values was observed, confirming that the composite of PAN and cellulose nanocrystals facilitates an increase in the planar zigzag sequence.

**Figure 3:**
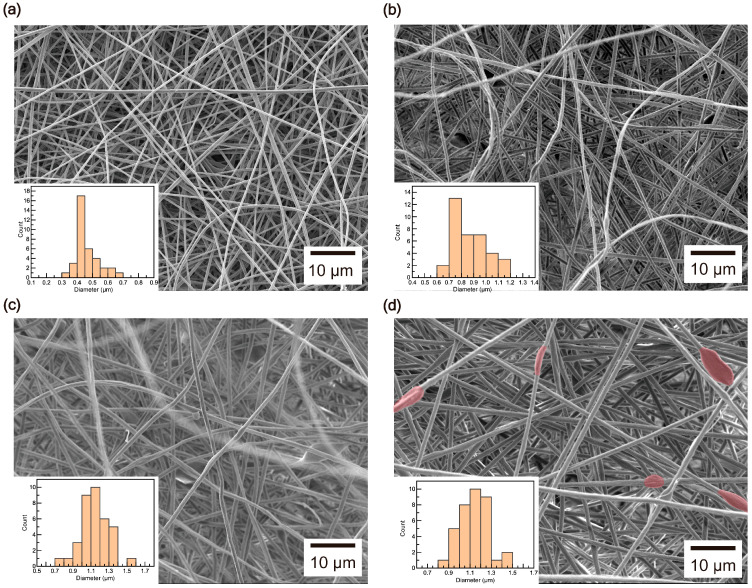
SEM images of (**a**) PAN, (**b**) PAN/CNC1, (**c**) PAN/CNC2, and (**d**) PAN/CNC3. The regions where beads formed in (**d**) are highlighted in red. (Inset: histogram of composite fiber diameters).

The SEM results reveal that the diameters of the composite fibers increased with the addition of CNCs. Diameter analysis shows that under the conditions described in the experimental section, PAN fibers exhibited a diameter range of 0.4–0.5 μm (Figure 3a). However, as the CNC wt% increased, the diameters expanded to 0.7–1 μm at 1 wt% (Figure 3b), 1–1.2 μm at 2 wt% (Figure 3c), and 1.1–1.3 μm at 3 wt% (Figure 3d). This indicates that the fiber thickness became greater with higher CNC content. Furthermore, the addition of CNCs led to the formation of cellulose nanocrystal beads within the PAN fibers, creating a composite structure. Bead formations, which were not observed up to 2 wt%, became visible in multiple areas at 3 wt% (Figure 3d).

**Figure 4:**
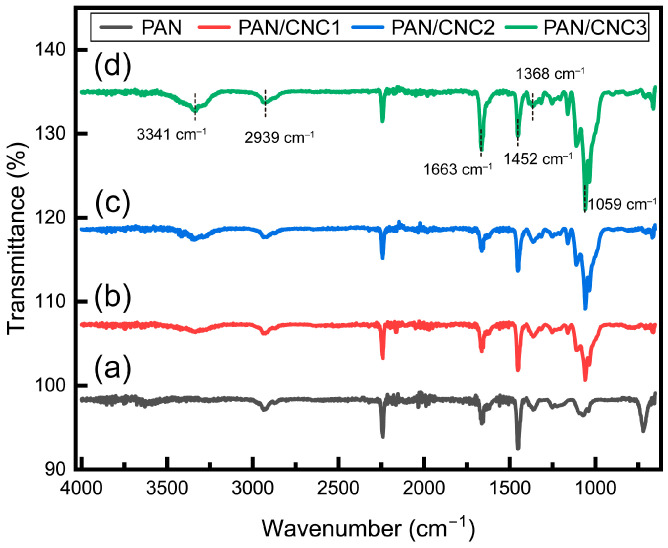
FTIR spectra for (**a**) PAN, (**b**) PAN/CNC1, (**c**) PAN/CNC2, and (**d**) PAN/CNC3.

As shown in Figure 4, FTIR analysis reveals the presence of a peak at 2240–2244 cm^−1^, corresponding to the CN nitrile group in the PAN structure. Peaks at 2937–2939 cm^−1^, 1449–1452 cm^−1^, 1363–1368 cm^−1^, and 720 cm^−1^ are attributed to the aliphatic CH group [29,30,31]. With the addition of CNCs, a peak was observed at 3200–3540 cm^−1^ due to O-H stretching, and a peak at 1663 cm^−1^ appeared due to O-H bending of absorbed water, with its intensity increasing as the CNC wt% increased. Additionally, peaks at 1059 cm^−1^ (C-OH stretching) also intensified with increasing CNC wt% [32]. The increase in the O-H peak of CNC at 3200–3540 cm^−1^ and at 1059 cm^−1^ confirms that the formation of composite fibers was promoted as the CNC wt% increased, demonstrating the successful formation of the PAN–CNC composite.

The increase in planar zigzag structure can also be confirmed through FTIR by analyzing the peak areas. Specifically, it can be calculated based on the vibration bands at 1250 cm^−1^ and 1230 cm^−1^. The formation of planar zigzag structures can be assessed by analyzing the area ratio of these two vibration bands. An increase in the area ratio at 1250 cm^−1^ indicates the enhancement of planar zigzag structures. This structural configuration reduces the C-C bond distance, leading to a squeeze effect from -CN nitrogen, which subsequently enhances the electrical properties. The calculation is performed using the following equation [12]:

(1) $$\phi =\frac{S_{1250}}{S_{1250}+S_{1230}}$$

**Figure 5:**
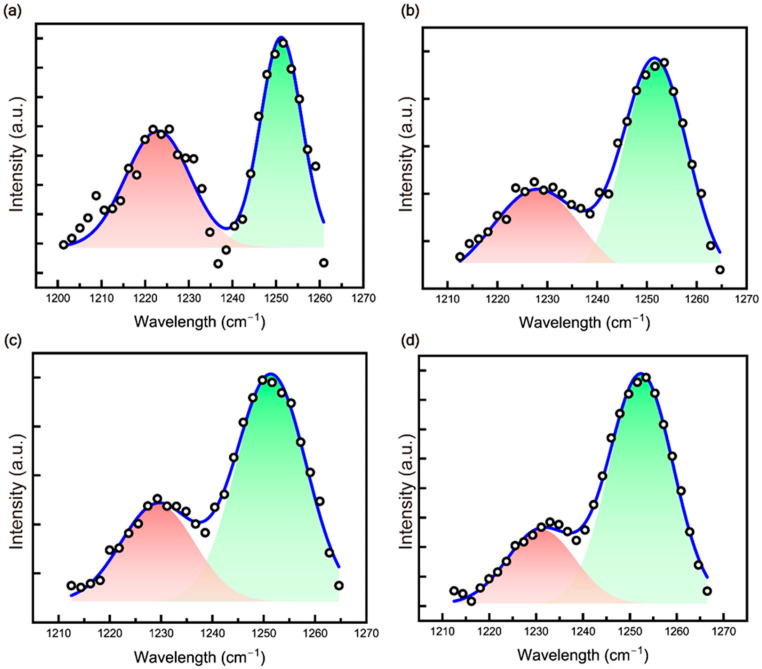
FTIR spectra in the range of 1210–1270 cm^−1^ for (**a**) PAN, (**b**) PAN/CNC1, (**c**) PAN/CNC2, and (**d**) PAN/CNC3.

Equation (1) shows that *S*_1230_ and *S*_1250_ represent the peak areas at 1230 cm^−1^ and 1250 cm^−1^, respectively. The area at 1250 cm^−1^ is used to calculate the proportion of the zigzag conformation, while the total area includes the contribution from the 3^1^-helical structure at 1230 cm^−1^. This equation determines the ratio of the zigzag conformation within the overall structure. A higher peak area ratio at 1250 cm^−1^ indicates a greater formation of planar zigzag structures. Comparing the peak areas at this wavenumber for composite fibers reveals that PAN showed 53.51%, PAN/CNC1 yielded 63.59%, PAN/CNC2 exhibited 68.85%, and PAN/CNC3 reached 74.27%. These results confirm that the planar zigzag structure increased as the CNC wt% increased (Figure 5).

The increase in planar zigzag structures with CNC addition is attributed to the well-aligned CNCs promoting the alignment of PAN chain orientations [19]. In this study, through the electrospinning method, we not only demonstrated the improvement in mechanical properties due to CNC addition to PAN but also, for the first time, analyzed and demonstrated the enhancement of piezoelectric properties and the formation of zigzag structures through XRD, electrical characteristics, and FTIR analyses.

**Figure 6:**
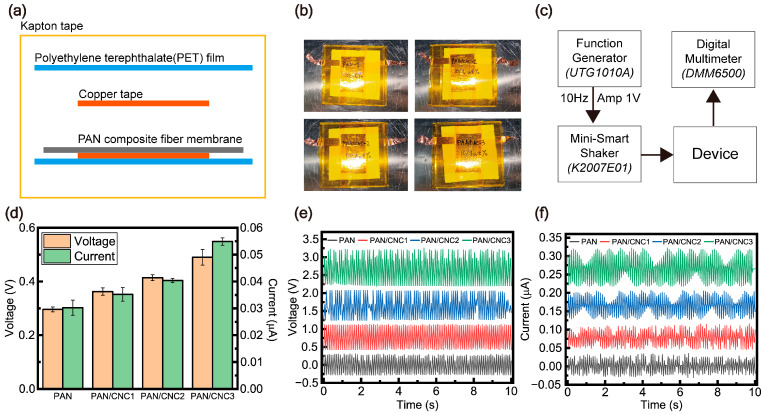
(**a**) Schematic of device structure. (**b**) Piezoelectric device. (**c**) Custom-built setup for electrical signal measurement. (**d**) Comparison of electrical characteristics. (**e**) Voltage characteristics. (**f**) Current characteristics.

Using the fabricated device, we applied a force at 10 Hz through a shaker produced consistent electrical signals, allowing for the measurement of voltage and current (Figure 6). The force applied to the device using the shaker was measured to be 7.4 N. A device was fabricated using copper tape as electrodes on the PAN composite membrane and encapsulated with a PET film (Figure 6a,b). As described in Section 2.5 on characterization, the electrical properties of the fabricated device were evaluated using a function generator, a shaker, and a digital multimeter (Figure 6c). A comparison of the average and standard deviation of the piezoelectric performance indicates that the enhancement in piezoelectric properties is attributed to the planar zigzag structure (Figure 6d). Notably, both the experimentally measured voltage and current increased, confirming that molecular structural changed significantly contribute to the improvement in electrical properties. Additionally, the strong charge separation characteristic of piezoelectric materials resulted in a simultaneous increase in positive and negative charge generation (Figure 6e,f). It was observed that the voltage and current increased with higher CNC content. In the case of pristine PAN material, piezoelectric properties of 0.296 V and 0.03 µA were observed (Figure 6c). With the addition of CNCs, these properties were found to improve, reaching 0.49 V and 0.054 µA at 3 wt% CNC content. This increase can be attributed to the structural changes in PAN, specifically the enhanced planar zigzag structure confirmed through XRD and FTIR analyses. The increase in the planar zigzag structure indicates a reduction in the C–C bond distance within the PAN polymer chain, which influences the molecular structure of the polymer [28]. Specifically, in the planar structure, the C-C bond distance is shorter than in the helical structure, causing the electron cloud to shift toward the carbon atoms. This shift leads to the squeeze effect of the -CN nitrogen atoms, resulting in a chemical shift of the carbon [12]. Consequently, this structural transformation enhances the electrical properties of PAN, ultimately improving its piezoelectric performance. Thus, the addition of CNC was shown to improve the electrical properties of the device. Since the planar zigzag structure can be easily controlled by adjusting CNC content, this material holds immense potential for future applications in novel piezoelectric devices. (Table 1) Furthermore, to expand the application of PAN-CNC composites across various industrial fields, additional research on device optimization and green composites is required.

## 4. Conclusions

In this study, for the first time, a PAN–cellulose nanocrystal composite was fabricated using the electrospinning method. By incorporating CNCs, a novel approach was successfully developed to enhance the content of planar zigzag structures, and a piezoelectric device was produced to confirm improved piezoelectric properties. Structural changes in the PAN–cellulose nanocrystal polymer composite, including variations in nanofibers and an increase in planar zigzag formations, were verified through XRD and FTIR analyses. The enhancement in electrical characteristics was observed under loading conditions applied via a miniature shaker. When CNC content was increased up to 3 wt%, voltage showed a 68.9% improvement, and current increased by approximately 80% compared to pristine PAN. This demonstrates that the addition of CNC to PAN contributes to the formation of zigzag structures, leading to mechanical property enhancements and improved piezoelectric performance. The novel PAN–cellulose nanocrystal material, as a green composite, holds significant potential for applications in wearable sensors and nanogenerators utilizing human-friendly components.

## Figures and Tables

**Table 1:** Results of the piezoelectric properties based on the weight percent of CNCs in the PAN composite material.

PAN (wt%)	CNC (wt%)	Voltage (V)	Current (µA)	Zigzag Conformation (%)
10	0	0.296 ± 0.008	0.03 ± 0.002	53.51
	1	0.362 ± 0.013	0.035 ± 0.002	63.59
	2	0.414 ± 0.01	0.04 ± 0.0007	68.85
	3	0.49 ± 0.028	0.054 ± 0.001	74.27

## Data Availability

The data presented in this study are available in the insert article.
